# A Sarcopenia Detection System Using an RGB-D Camera and an Ultrasound Probe: Eye-in-Hand Approach

**DOI:** 10.3390/bios11070243

**Published:** 2021-07-16

**Authors:** Yeoun-Jae Kim, Jueun Choi, Jungwoo Moon, Kyung Rim Sung, Jaesoon Choi

**Affiliations:** 1 Biomedical Engineering Research Center, Asan Institute for Life Sciences, Asan Medical Center, 88, Olympic-Ro 43-Gil, Songpa-Gu, Seoul 05505, Korea; lethkim1@gmail.com; 2 Department of Biomedical Engineering, Asan Medical Center, University of Ulsan College of Medicine, Seoul 05505, Korea; cje20205392@mail.ulsan.ac.kr (J.C.); mjw0592@mail.ulsan.ac.kr (J.M.); 3 Department of Ophthalmology, Asan Medical Center, University of Ulsan College of Medicine, Seoul 05505, Korea

**Keywords:** sarcopenia detection, ultrasound scanning, RGB-D camera, eye in hand, in-vitro test, bean curd-gelatine phantom, muscle thickness measurement

## Abstract

Skeletal muscle mass deficiency and quality degradation constitute sarcopenia for elderly people. Sarcopenia can result in musculoskeletal damage and accompany various metabolic problems, which make early sarcopenia diagnosis important. Various modalities, such as computed tomography (CT) and magnetic resonance imaging (MRI), have been developed for screening sarcopenia. Recently, ultrasound scanning was suggested for screening sarcopenia because of its safety, usability, and cost effectiveness. However, there has been no standardized assessment methodology for screening sarcopenia with ultrasound scanning. Therefore, prior to this study, we developed a four-degrees-of-freedom (DOF) sarcopenia detection system using an RGB-D camera and an ultrasound probe to automatically scan the human thigh without operator dependency. However, due to the eye-to-hand approach with the RGB-D camera, the system has limited usability for clinical trials. Therefore, in this study we modified the system such that it became eye-in-hand by attaching the RGB-D camera to the upper part of the system with an enhanced arc fitting algorithm. The modified system and enhanced algorithm were verified by an in-vitro test with bean curd-gelatin phantom. The results showed that the thickness of bean curd in the gelatin phantom was maintained at approximately 12.7 ± 0.35 mm over the 71.5${}^{\circ }$ scanning range with 2.49 ± 0.15 N radial force at various thickness measuring points.

## 1. Introduction

Sarcopenia is characterized by muscle mass deficiency and quality degradation [1], which can induce decline of musculoskeletal function and impaired mobility. Moreover, it is accompanied by metabolic problem such as obesity [2]. In US, approximately 45% of order people are sarcopenic [3]. For screening during the early stages of sarcopenia, various modalities have been suggested and applied for clinical usage, such as anthropometry [4,5,6], bioelectrical impedance analysis (BIA) [7,8,9], dual X-ray absorptiometry (DEXA) [10], CT/MRI [11,12,13], and sonography [14,15,16,17,18,19,20,21,22,23]. For a recent comprehensive survey of sarcopenia screening with artificial intelligence using CT/MRI, refer to [13].

Among these various modalities, sonography has recently been promoted for sarcopenia screening because of its safety, usability, and cost effectiveness compared with other modalities. However, only a few non-commercial systems have been developed worldwide to address the possibility of ultrasound scanning for sarcopenia detection. For the ultrasound modality for sarcopenia screening, various assessment sites (lower limb, rectus femoris, Quadriceps femoris, etc.) and parameters (muscle thickness, muscle cross-sectional area, echo intensity, pennation angle, elastography, etc.) [14] are used. For the various biomarkers used in sarcopenia assessments in clinical situations, refer to [14].

Among those parameters for sarcopenia screening with sonography, muscle thickness measurement is a quantitative means to detect sarcopenia with level of evidence 2 [14]. Compared with other parameters, it is relatively easy to quantize and correlate in sarcopenia screening. There are several papers that report using muscle thickness for sarcopenia detection [15,16,17,18,19,20,21,22,23,24,25,26].

For muscle thickness measurements while seeking data correlations in sarcopenia screening, Wang et al. [15] measured the muscle thicknesses (MT), fat thicknesses (FT), and MT/body mass indexes (BMI) of one hundred thirty-five elderly participants, which were used to analyze the correlation between ultrasound (US) measurements of the gastrocnemius muscle and low muscle mass, as defined by sarcopenia. Hida et al. [16] compared thigh muscle thickness in ultrasound images with BIA. Strasser el al. [17] measured muscle thickness using the pennation angle in the quadriceps in neuromuscularly unimpaired patients. Minetto et al. [18] compared the muscle thicknesses and masses of the quadriceps group from ultrasound images.

Additionally, Rustani et al. [19] performed rectus femoris muscle (RFM) thickness measurements on 119 patients by ultrasound B-mode scanning and suggested that RFM is an appropriate muscle for sarcopenia screening in the elderly. Zhu et al. [20] measured the MT of both the forearm and the lower leg and the pennation angle of the gastrocnemius in a total of 265 elderly Chinese community dwellers. They concluded that a combination of posterior tibial MT and anterior ulnar MT measured by muscle ultrasound is helpful for sarcopenia screening in elderly Chinese men. Salim et al. [21] performed thigh muscle thickness measurements and normalized them to patient thigh length. They used 49 patients, and concluded that the normalized muscle thickness index can be used as a surrogate to a CT scan, whereby it can identify elderly frail patients with sarcopenia.

However, one of the difficult parts of using ultrasound scanning to diagnose sarcopenia is its operator dependency. Since the orientation and interfacing pressure between the ultrasound probe and the subject’s skin surface are different with respect to the operator [22], it is hard to accurately quantize the muscle thickness and muscle area. To accurately quantify the muscle thickness and muscle area from an ultrasound image in sarcopenia screening, as described by the above references, the ultrasound probe must be accurately oriented with regard to the surface of skin and the probe pushing force must be maintained at a predefined level during scanning. For the quantitative ultrasound, Harris-Love et al. [23,24] performed feedback force-augmented quantitative ultrasound phantom tests with the KUKA robot. In addition, Correa-de-Araujo and Harris-Love et al. [25] published a symposium report regarding a standardized assessment of muscle quality in skeletal muscle function and dysfunction. In the report, the sonographer and force variations in quantitative muscle sonography were summarized. However, these were preliminary works for quantization of ultrasound modality, not the qualitative development of a specific ultrasound device.

To solve problems in the field, we developed a four-DOF sarcopenia detection system with a commercial ultrasound probe, an RGB-D camera, and a force sensor to accurately scan subjects’ thighs [26]. The method proposed in [26] scans the subject’s thigh with the RGB-D camera and arc fits the point cloud of the surface fo the subject’s thigh to continuously orient the ultrasound probe normally with respect to the surface and scan the subject’s thigh angularly while maintaining a predefined amount of pressure. The clinician can investigate the ultrasound image sequences during scanning and make use of the ultrasound image information for diagnosing sarcopenia.

To validate the developed system, the in-vitro angular scanning test was performed to investigate whether there are tissue thickness changes during scanning. If the tissue thickness changes are minimized during angular scanning, the above requirements (ultrasound probe orientation and contact force) of operator-independent thigh scanning will be considered fulfilled. The result of an in-vitro test in the previous work [26] was 26.01 ± 1.0 mm for average ham (meat) tissue thickness during 82${}^{\circ }$ of scanning with 2.5 N of radial force, which is a promising result.

However, because the RGB-D camera is located outside of the system (eye-to-hand approach, Figure 3a,b in [26]), it is inconvenient for real clinical situations (the camera tripod disturbs the patient). The position/orientation of the camera is kept unchanged for camera calibration accuracy. Therefore, the authors changed the previously developed system from being eye-to-hand to eye-in-hand by attaching the RGB-D camera to the upper part of the system with an enhanced arc fitting algorithm. By changing the system to an eye-in-hand approach, it can be compact and practical in clinical situations. However, in the eye-in-hand approach, the RGB-D camera moves with respect to the angular movement of the developed system, which makes the robot–camera coordination alignment and arc fitting algorithm different from those of the eye-to-hand system configuration.

The main improvements we present in comparison to our previous work [26] are as follows.

1. The eye-to-hand configuration of the RGB-D camera was changed to an eye-in-hand configuration for clinical feasibility. The RGB-D camera was also changed with respect to [27,28] to gather denser point clouds of subjects’ thigh surfaces.
2. The arc curve fitting method of the angular surface of the subject’s thigh with an RGB-D camera with piecewise arcs [26] was changed to accommodate the eye-in-hand configuration. Moreover, in the proposed method, algebraic and geometric fitting methods [29,30,31,32] are both used to render the curve fitting result more quickly than the previous method (enhanced piecewise arc curve fitting method).
3. An in-vitro test with bean curd-gelatin phantom was performed to validate the system and the proposed method. In opposition to the single-point muscle thickness measurements using ultrasound images in previous work [26], multiple-point bean curd thickness was measured.

Section 2 overviews the developed sarcopenia detection system and the proposed angular thigh scanning method, with emphasis on the enhancements. The components that remained unchanged from the previous work [26] are minimally mentioned, but specific references are given for completeness of the paper. Section 3 presents the bean curd-gelatin phantom test. The research summary and future research directions are in the Section 4.

## 2. Materials and Methods

### 2.1. The Modified Sarcopenia Detection System

**Figure 1:**
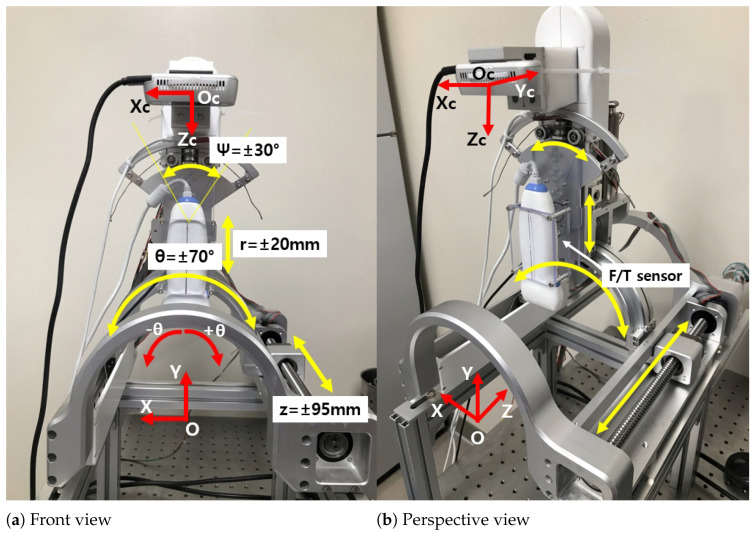
Overview of the modified 4-DOF sarcopenia detection system.

The front and perspective views of the modified sarcopenia detection system are represented in Figure 1a,b, respectively. As explained in the introduction, the RGB-D camera (Intel Realsense D435i [28]) was attached to the upper part of the original system to create an eye-in-hand configuration. The camera has a range of 0.3∼3.0 m; RGB and depth fields of view (FOV) are 69${}^{\circ }$× 42${}^{\circ }$ and 87${}^{\circ }$× 58${}^{\circ }$, respectively. RGB and depth image resolutions are 1920 × 1080 @30 fps and 1280 × 720 @90 fps. Accuracy of depth is <2% @2 m.

The two red coordinate systems in Figure 1a,b represent camera coordinates and robot coordinates, respectively. Note that the subscript “c” represents the camera. The four yellow arrows in Figure 1a,b indicate the four-DOF movement of the modified system, just as the previous system had [26]. Variables z, r, θ and ψ in Figure 1a represent z and y directional linear movements and two angular movements. The F/T sensor in Figure 1b is attached to the upper part of the system to measure the ultrasound probe’s contact force, as in the previous system. Note that the RGB-D camera moves with respect to θ directional movement for eye-in-hand configuration (Figure 1a,b) and +θ direction is clockwise from vertical. For more details of the sarcopenia detection system and communications, see Section 2.1 in [26].

### 2.2. Modified Overall Control Flow Diagram

When a subject’s thigh is inserted to the arch-shaped lower part of the system in Figure 1a,b, the ultrasound probe automatically scans the surface of the thigh by planar angular thigh scanning method, which is proposed in Section 2.2 of [26]. The dimensions, type, depth range, bandwidth, and patient contact area of the ultrasound probe are 142 mm × 58 mm, flat, 1∼100 mm, 5∼10 MHz and 38 mm, respectively. The angular thigh scanning method uses a piecewise arc curve fitting method (Section 2.2.1 of [26]) and an ultrasound probe moving method (Section 2.2.2 in [26]), which includes planar kinematics and a Jacobian-based probe moving method. However, one of the major shortcomings of the angular thigh scanning method we used previously is that it takes time to finish the piecewise arc curve fitting, too long for efficient diagnosis. Therefore, an enhanced piecewise arc curve fitting method is proposed. The proposed method combines algebraic and geometric circle fitting methods to maximize performance and minimize the calculation time.

**Figure 2:**
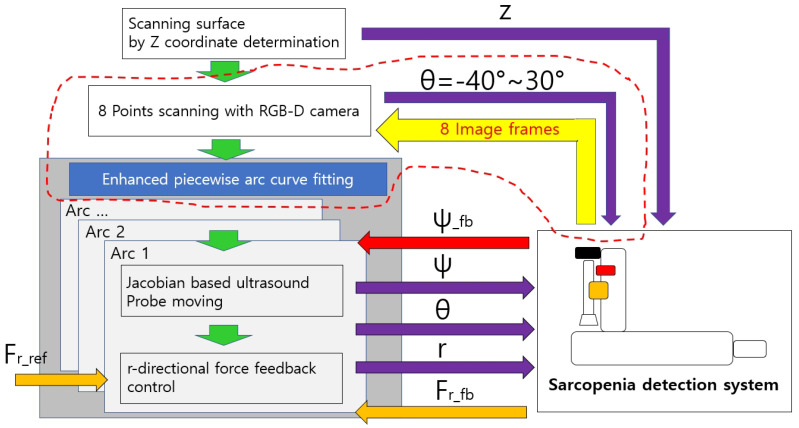
The modified overall control flow diagram (the parts modified from previous work [26] are surrounded by the red dotted line).

Figure 2 represents the modified overall control flow diagram of the modified sarcopenia detection system. The changed parts from the previous work are surrounded by the red dotted line in Figure 2. In Figure 2, after z directional scanning surface determination and movement by the linear guide in Figure 1a,b, 8-point scanning with the RGB-D camera is performed to gather point clouds by moving the camera in θ direction (θ = −40${}^{\circ }$, −30${}^{\circ }$, −20${}^{\circ }$, −10${}^{\circ }$, 0${}^{\circ }$, 10${}^{\circ }$, 20${}^{\circ }$, 30${}^{\circ }$) as represented in Figure 9b–i. Then, eight point clouds are gathered at each scanning angle, which are depicted as red arcs in Figure 9b–i.

The curve-fitted arcs are generated by the proposed enhanced piecewise arc curve fitting method to best fit the gathered point clouds. Each fitted arc is scanned by the proposed Jacobian-based ultrasound probe moving method (θ and ψ angles in Figure 1a) and r directional force feedback control (r in Figure 1a). Note that r and θ in Figure 2 are calibrated beforehand for positional control in each direction. ψ and F${}_{r}$ (radial force) are feedback controlled.

### 2.3. The Enhanced Piecewise Arc Curve Fitting Method

#### 2.3.1. Details of the Enhanced Piecewise Arc Curve Fitting Method

**Figure 3:**
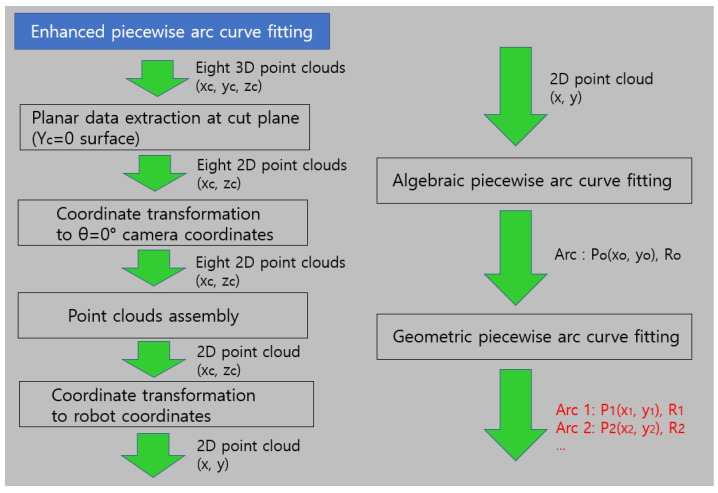
The enhanced piecewise arc curve fitting method.

The 1-point scanning of the previous work was changed to 8-point scanning with the RGB-D camera to gather more point clouds from different points of view, as depicted in Figure 9b–i. The enhanced piecewire arc curve fitting method located in the gray rectangle in Figure 2 is represented in detail in Figure 3.

The enhanced piecewise arc fitting method consists of six parts, which are six rectangles in Figure 3. The input and output of each part are represented on the right side of the green arrow. Note that x${}_{c}$, y${}_{c}$ and z${}_{c}$ in Figure 3 represent point data in camera coordinates; and x, y and z denote the same in robot coordinates. The four rectangles on the left in Figure 3 perform coordinate transformations of the eight 3D point clouds to 2D point clouds in robot coordinates. As the point cloud acquired at each θ angle is in camera coordinates, the eight point clouds in Figure 9b–i first are converted to θ = 0${}^{\circ }$ camera coordinates and then combined into one file. Then, the point combined cloud in the file is converted to robot coordinates via transformation matrix.

With the assembled point cloud, algebraic piecewise arc curve fitting [29] is performed using Pratt’s method [30] as depicted on the right side of Figure 3. The outputs of the algebraic piecewise curve fitting are the center points of arc (P(x, y)) and the radius of arc (R). With the algebraic fitting results, geometric piecewise arc curve fitting [31,32] is performed using the steepest descent algorithm [32] to render the piecewise arcs ((P${}_{1}$(x${}_{o}$, y${}_{o}$), P${}_{2}$(x${}_{o}$, y${}_{o}$), …) and (R${}_{1}$, R${}_{2}$, …)). Details of geometric piecewise arc curve fitting are in Appendix A.

**Figure 4:**
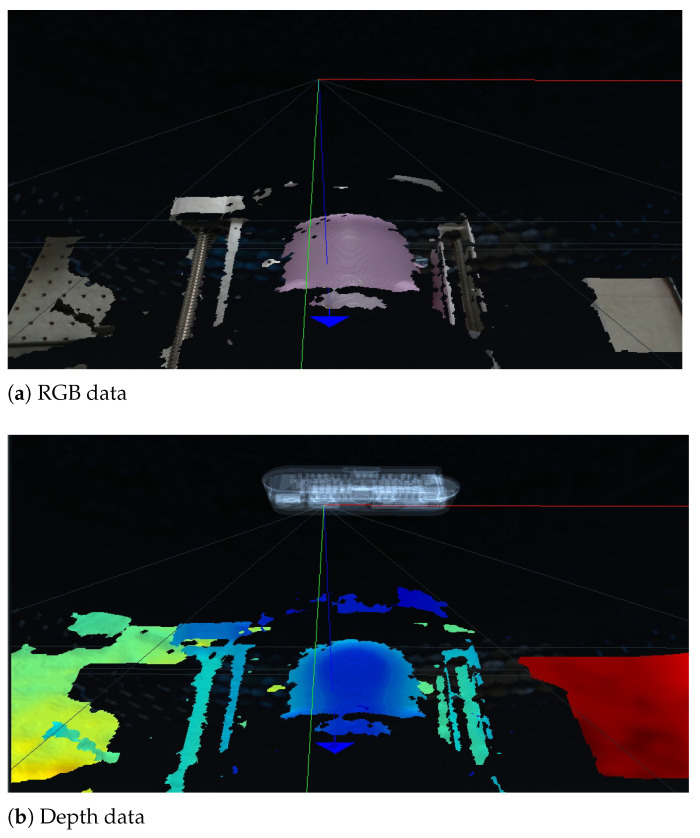
A point cloud example using an RGB-D camera and API [28,33].

**Figure 5:**
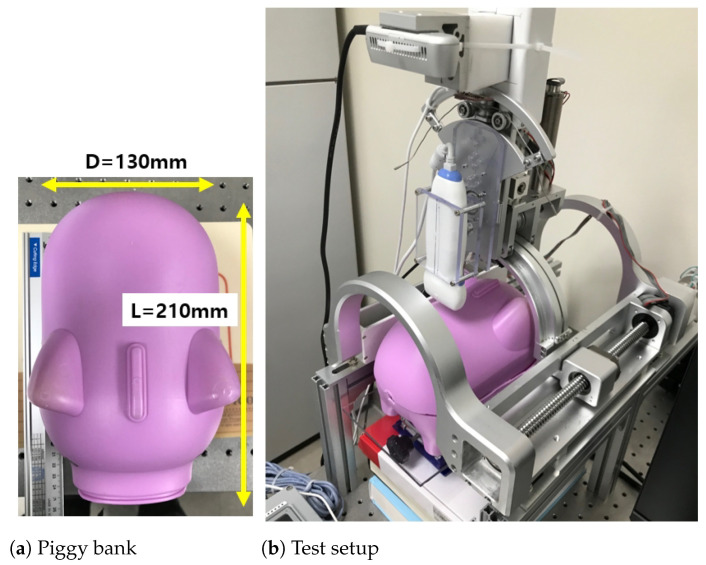
The enhanced piecewise arc curve fitting test setup.

A point cloud example with an RGB-D camera [28] is in Figure 4a,b, which represents the back of a piggy bank in Figure 5a,b. The point cloud data were extracted from the camera vendor’s program and application programming interface (API) [33]. To verify the enhanced piecewise arc curve fitting method in Figure 3, the piggy bank in Figure 5a was inserted in the lower part of the sarcopenia detection system, which is depicted in Figure 5b. Then, the scanning surface by z coordinate determination, 8-point scanning with the RGB-D camera, and enhanced piecewise arc fitting were sequentially performed, as described on the left sides of Figure 2 and Figure 3.

**Figure 6:**
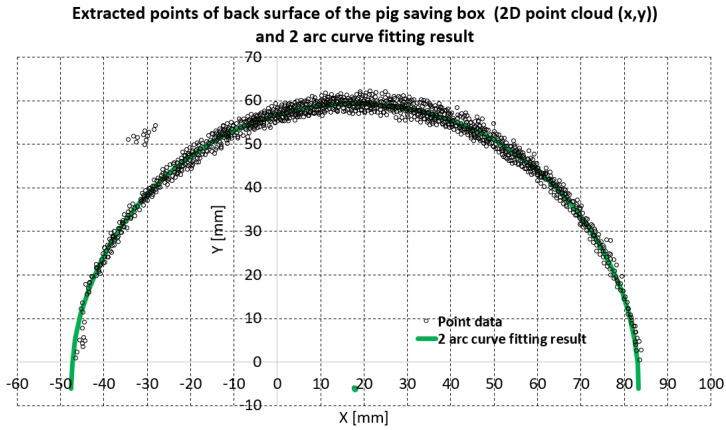
Piecewise curve fitting results of the back surface of the piggy bank (in total, 1164 points). The first and second arcs made via geometric method in Table 1 are almost the same; therefore, the two green arcs look like one arc.

The piecewise arc curve fitting result by the proposed method is in Table 1 and Figure 6. The total number of points extracted from the example in Figure 6 was 1164; there were 12 outlier points, as seen in the upper-left portion of Figure 6. The algebraic fitting result and geometric fitting result in Table 1 are almost the same: there is a less than 0.1 mm difference in center points and a less than 0.15 mm different in radius, which means that the acquired point cloud is very circular. The total error and average error in Table 1 were calculated by Equations (1) and (2), which are the same as Equations (1) and (2) in [26].

In Equations (1) and (2), $p_{ref\_ji}$ represents the reference points shown as point data in Figure 6. $p_{fitting\_ji}$ represents the resultant fitting points, which are the green fitting results in Figure 6. Note that *j* is arc number and *i* is the point number for each arc. In Equations (1) and (2), *M* and *N* are the total arc number and total point number in each arc. For Table 1 and Figure 6, *N* was set to 1164/2 = 582 (points) and *M* was set to 2. The total error and average error of geometric fitting (Table 1) were approximately 80.32 mm and 0.069 mm, respectively, which could be considered good arc fitting results for 1164 points.

(1) $$total\_error=\sqrt{\sum_{j=1}^{M}\sum_{i=1}^{N}{\left({∥p_{ref\_ji}-p_{fitting\_ji}∥}_{2}\right)}^{2}}$$

(2) $$average\_error=\sqrt{\frac{\sum_{j=1}^{M}\sum_{i=1}^{N}{\left({∥p_{ref\_ji}-p_{fitting\_ji}∥}_{2}\right)}^{2}}{MN}}$$

The green arc in Figure 6 represents the geometric fitting result overlaid on the 1164 extracted points. Note that the green dot in Figure 6 represents the center of the green arc, and the algebraic fitting result is omitted in Figure 6 because it is almost the same as the geometric fitting result. The point cloud in Figure 6 is an approximately ±3∼4 mm thick band from the green arc, which agrees with accuracy of the depth of the RGB-D camera [28].

#### 2.3.2. A Comparison of the Enhanced Piecewise Arc Curve Fitting with the Piecewise Arc Curve Fitting

The results of the piecewise arc curve fitting method of [26] are compared with those of the proposed enhanced piecewise arc curve fitting method, using the same data points from Figure 9b in [26], a total of 32 points. That is far fewer points than the 1164 points in Figure 6 because of the RGB-D camera [26,27] and the scanning methods (single shot scanning vs. 8-point scanning) are different. Additionally, the piggy bank’s position in the sarcopenia detection system during the experiment was different in [26] than during this study (Figure 5b).

Both methods were run on the main PC (Intel(R) Core(TM) i7-3930K CPU, 16.0GB RAM, Windows 10 Enterprise) and programmed with Visual Studio 2019 C++ compiler/linker. The results are summarized in Table 2.

As presented in Table 2, the total error and average error of the proposed method were approximately 1.16 (=2.38–1.22) mm and 0.03 (=0.07–0.04) mm larger than those of the method in [26]. However, the elapsed time of the proposed method was 9 m, which is incomparable to the 9475 ms of the method presented in [26]. In clinical a situation, the 0.03 mm error difference in Table 2 is trivial compared with a 9.466 s time difference.

## 3. Results

In the previous work, the developed system and method was validated via in-vitro ham-gelatin phantom, which showed 26.01 ± 1.0 mm average ham tissue thickness during 82${}^{\circ }$ of scanning with 2.5 N radial force. In this work, the modified system with 8-point scanning and the enhanced piecewise arc curve fitting method were validated by in-vitro testing with a bean curd-gelatin phantom.

### 3.1. Bean Curd-Gelatin Phantom

**Figure 7:**
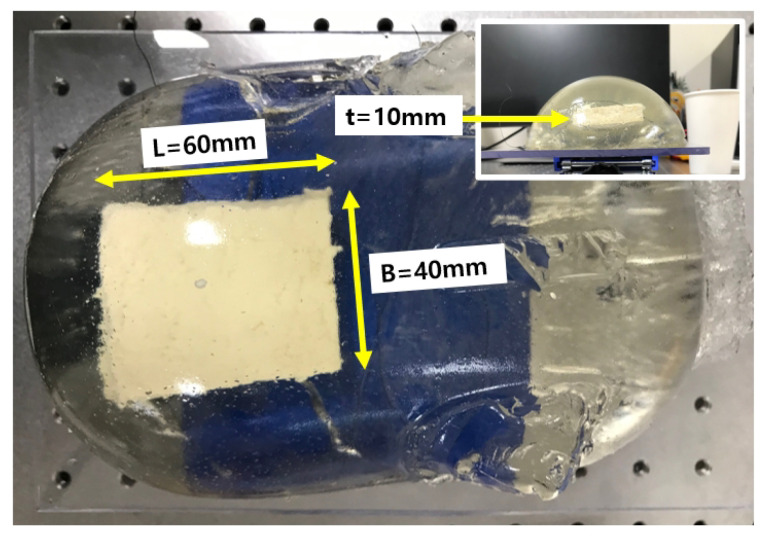
Bean curd-gelatin phantom (L: length, B: width, t: thickness).

The bean curd-gelatin phantom is depicted in Figure 7. It was a rectangular parallelpiped approximately 60 × 40 × 10 mm in size, immersed in a 3% gelatin phantom [26], which mimics the stiffness of the human thigh. The composition of the bean curd was 100% soybeans. The piggy bank in Figure 5a was used as a mold for the phantom’s manufacturing.

### 3.2. In-Vitro Bean Curd-Gelatin Phantom Test Results

**Figure 8:**
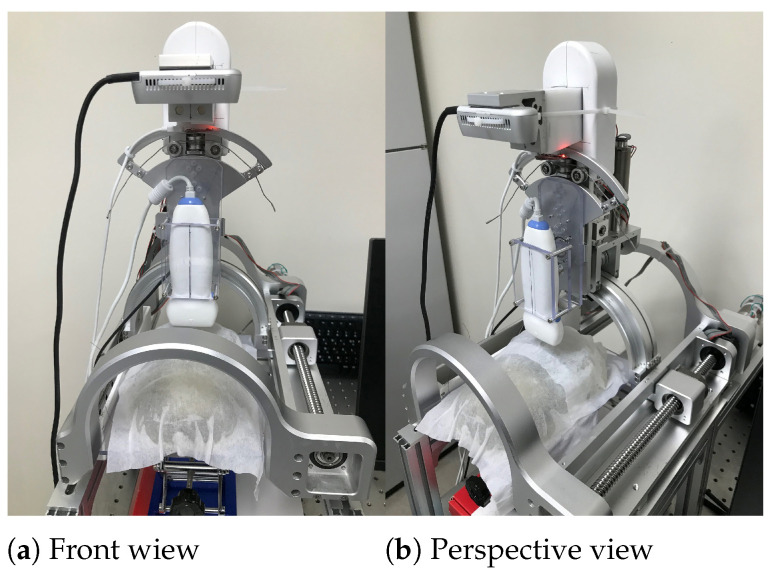
In-vitro test pictures.

**Figure 9:**
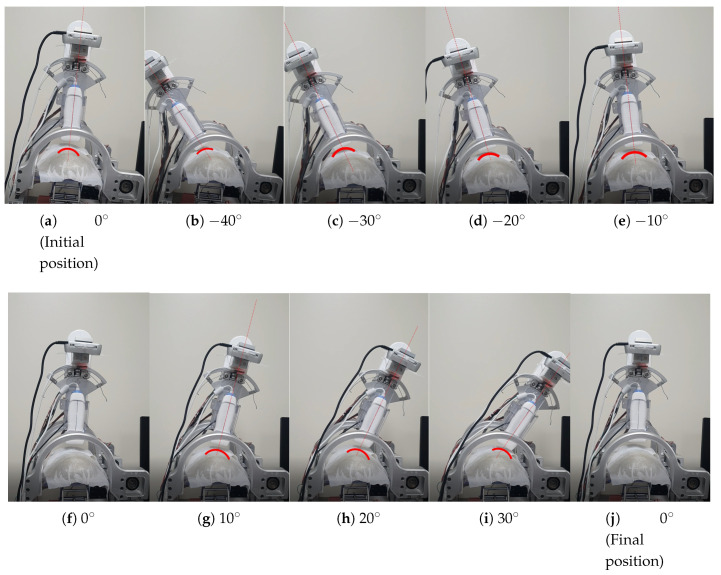
Eight-point scanning snapshots (the red arcs in (**a**–**i**) represent the point cloud acquired by scanning).

**Figure 10:**
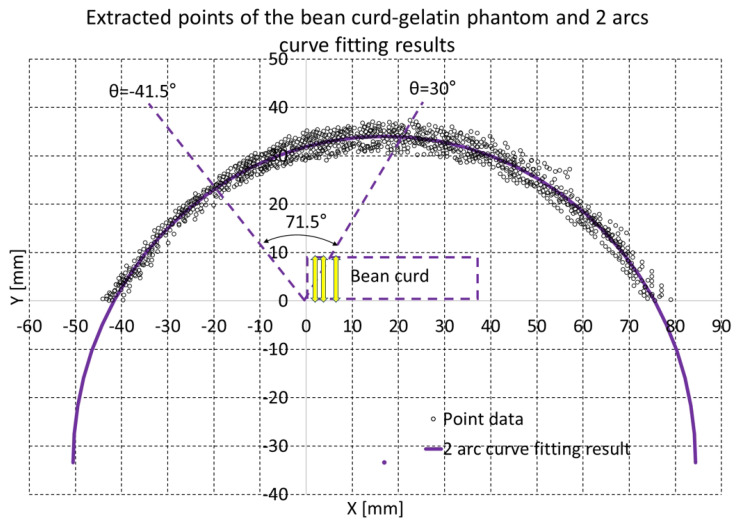
Enhanced piecewise curve fitting results of the bean-curd gelatin phantom (in total, 985 points).

The bean curd-gelatin phantom was placed under the lower part of the sarcopenia detection system, as depicted in Figure 8a,b. Note that because the bean-curd gelatin was transparent, a wet tissue was placed on it allow proper scanning by the RGB-D camera. The in-vitro test was performed by following the procedures in Figure 2 and Figure 3. The 8-point scanning in Figure 2 is depicted in Figure 9a–j, which shows that the sarcopenia detection system first moved from the initial position (θ = 0${}^{\circ }$) to θ = −40${}^{\circ }$, and then scanning began. After scanning at θ = 30${}^{\circ }$, the system returned to the initial position. The two arc curve fitting results are represented in Figure 10 and Table 3. In total, 985 points were collected in the feasible range.

Regarding the fitting results in Table 1, the center point and radius of the algebraic arc and geometric first and second arcs in Table 3 are almost the same, having only minor differences. The radius of the bean-curd gelatin phantom was approximately 67.5 mm, slightly larger than the 65.5 mm piggy bank in Table 1, which was probably due to the effect of the gelatin phantom’s weight and the wet tissue. Additionally, the y coordinate of the center point in Table 3 is approximately −33.5 mm, far lower than the −6.1 mm of the piggy bank shown in Table 1, which means that the bean curd gelatin phantom was placed lower than the piggy bank.

**Figure 11:**
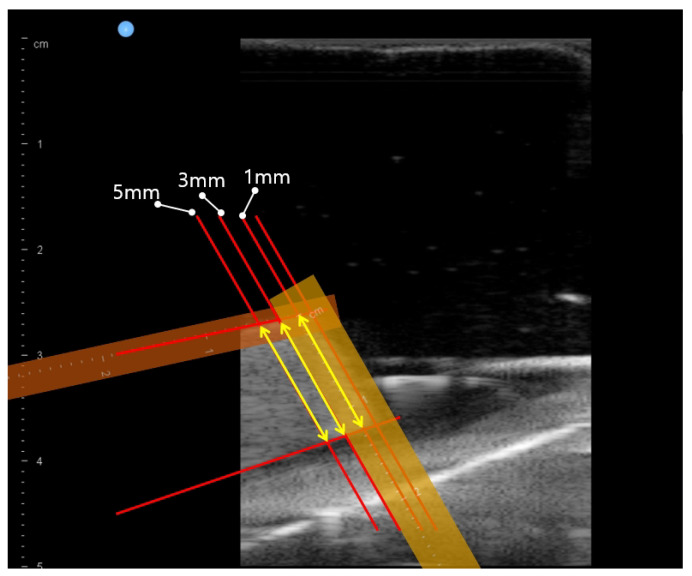
Thickness measurement positions at θ = −40${}^{\circ }$ (yellow arrows represent thickness 1, 3 and 5 mm from the upper right corner of the bean curd as depicted in Figure 10).

In Figure 10, the violet arc represents the geometric arc fitting results. The two dashed lines in Figure 10 represent the 71.5${}^{\circ }$ scan range (−41.5${}^{\circ }$∼30${}^{\circ }$), and the dashed rectangle represents the approximate bean curd position. The three yellow arrows in the dashed rectangle represent thickness 1, 3 and 5 mm from the upper right corner, which are also depicted in Figure 11. The points on the right-hand side in Figure 10 are rather scattered, due to the surface of the wet tissue, which as depicted in Figure 8a,b, was not uniformly placed on the back of the bean curd-gelatin phantom.

**Figure 12:**
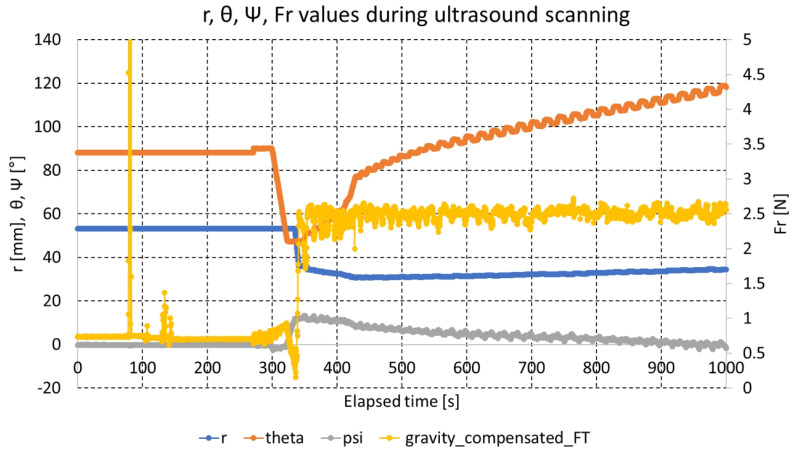
r, θ, ψ, and Fr measurements during ultrasound scanning.

Figure 12 presents r, θ and ψ, gravity compensated radial force measurements during ultrasound scanning with the bean curd-gelatin phantom. The scanning began at approximately 300 s and ended after approximately 12 min. During the initial 300 s (5 min), 8-point scanning and enhanced piecewise arc curve fitting in Figure 2 were performed. After arc curve fitting, phantom scanning began. During scanning, the radial force Fr was maintained at 2.49 ± 0.15 N; it was set to 2.5 N. The θ -> ψ -> r control sequence was repeated at 0.5${}^{\circ }$θ value increments, and the ultrasound images sent to ultrasound probe vendor’s software were saved to files.

The three violet arrows in Figure 12 represent the first θ -> ψ -> r control sequence. The hunting in the θ graph is due to the delayed overshoot of θ measurement. ψ graph is also hunting according to the hunting of θ value.

After finishing the phantom scanning, the ultrasound images at θ = −40${}^{\circ }$, −30${}^{\circ }$, −20${}^{\circ }$, −10${}^{\circ }$, 0${}^{\circ }$, 10${}^{\circ }$, 20${}^{\circ }$ and 30${}^{\circ }$ scanning were extracted from the saved file. In each extracted image, the bean curd thickness was manually measured at 1, 3 and 5 mm positions from the upper right corner of the bean curd along the upper line, which is depicted in Figure 11 by three yellow arrows. These manual measurements were performed by five independent operators with the ultrasound probe vendor’s software.

**Figure 13:**
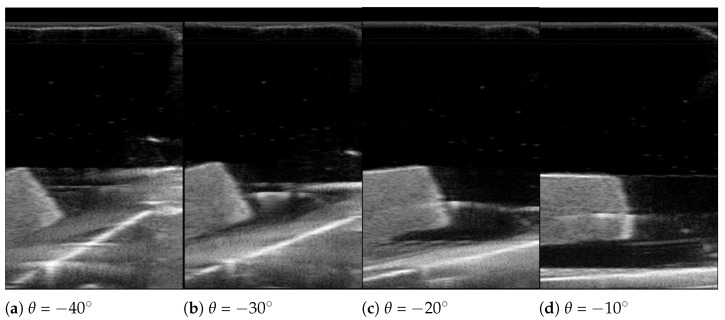

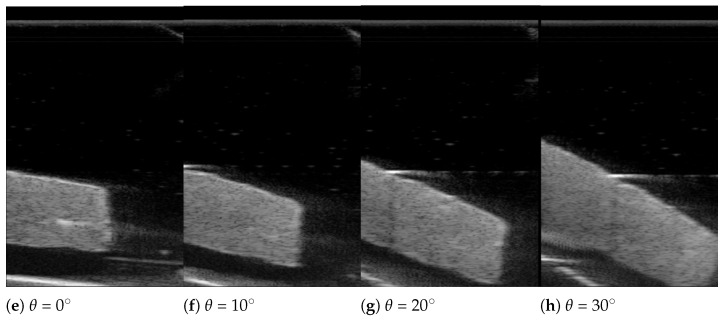
Images during ultrasound scanning.

The ultrasound scanning images are depicted in Figure 13 at θ = 50${}^{\circ }$–120${}^{\circ }$ and the bean curd thickness measurement results are in Table 4. In Figure 13a–h, the boundaries of the bean curd can be clearly identified, despite some scattering in the image. However, the bean curd’s entire cross-sectional area cannot be contained in a single image, because the width of the ultrasound images in Figure 13a–h is narrow compared with the width of the bean curd in the images.

The average thicknesses at 1, 3 and 5 mm positions were 12.58 ± 0.35 mm, 12.75 ± 0.34 mm and 12.77 ± 0.31 mm during 71.5${}^{\circ }$ scanning, as presented in the last row in Table 4. These results are thought to be superior to the 26.01 ± 1.0 mm during the 82${}^{\circ }$ scanning result of the previous study [26].

## 4. Discussion and Conclusions

In this study, the previously developed sarcopenia detection system, which had an eye-to-hand configuration, was modified to provide an eye-in-hand configuration with 8-point scanning and an enhanced piecewise arc curve fitting method. The eye-in-hand configuration can make the system compact and feasible for real clinical situations. The enhanced piecewise arc curve fitting method was compared with the previous method. The new method resulted in a far shorter running time (9.466 s decrease) and a slight increase in error (0.03 mm average error increase). The overall system was verified via an in-vitro bean curd-gelation phantom test. The test results showed that during 71.5${}^{\circ }$ of scanning, approximately ±0.35 mm of thickness deviation was present across an approximately 12.7 mm thick bean curd-gelatin phantom. The test was done with 2.49 ± 0.15 N radial force.

The result of the in-vitro test in the previous work was 26.01 ± 1.0 mm average ham tissue thickness during 82${}^{\circ }$ of scanning with 2.5 N of radial force. The present scan method is presumed to be superior to the previous scan method because the standard deviation decreased. However, the phantom and several settings were changed, so we cannot provide unequivocally superior results. In Figure 13a–h, the width of ultrasound images was too narrow to show the entire bean curd cross-sectional area in a single image. Therefore, we calculated the bean curd thickness only at 1, 3 and 5 mm positions from the upper right corner of the bean curd along the upper line in Figure 11. Future work will involve clinical trials with several subjects. Scaling of image width will be performed so that the thickness can be checked, and also the total cross-sectional area will be calculated. In future clinical trials, we should compare and verify the scan method presented here with the results of images manually scanned by clinicians.

Originally, the objective of the sarcopenia detection system with a eye-in-hand configuration was to help with diagnosing sarcopenia by robotizing the ultrasound scanning movement and acquiring standardized ultrasound images. By robotizing the ultrasound scanning movement, the operator dependency to the ultrasound image can be suppressed and more standardized ultrasound images can be acquired. In the current study, the interface pressure and the orientation of the ultrasound probe were thought to be properly controlled because the phantom thickness variations during the angular scanning process were small. However, as indicated in the Introduction, muscle thickness is one parameter among many other quantitative and qualitative parameters used to diagnose sarcopenia, and more synthetic protocols must be used [14]. Moreover, in the study, the thickness of the bean curd was manually measured by five independent operators. Automatic quantization of various parameters in the ultrasound image by image processing is desirable for the commercialization of the system.

## Figures and Tables

**Table 1:** Piecewise curve fitting result of back surface of the piggy bank (total 1164 points).

Fitting Name		Center Point		Radius	Error	
		X [mm]	Y [mm]	R [mm]	Total Error	Average Error
Algebraic	arc	17.839	−6.194	65.576	75.66	0.07
Geometric	1st arc	17.869	−6.114	65.436	80.32	0.069
	2nd arc	17.909	−6.114	65.466		

**Table 2:** A comparison of the enhanced piecewise arc curve fitting method with the piecewise arc curve fitting method from [26] (in total, 32 points).

Method Name		Center Point		R [mm]	Error [mm]	
		X [mm]	Y [mm]		Total Error	Average Error
Method in [26]	1st arc	−2.2	−56.89	73.62	1.22	0.04
	2nd arc	−5.79	−35.85	51.42		
Enhanced method	1st arc	−3.15	−50.92	70.11	2.38	0.07
	2nd arc	−7.41	−39.12	57.33		
Method name		Elapsed Time [ms]				
Method in [26]		9475				
Enhanced method		9				

**Table 3:** Piecewise curve fitting results of the bean-curd gelatin phantom (in total, 985 points).

Fitting Name		Center Point		Radius	Error	
		X [mm]	Y [mm]	R [mm]	Total Error	Average Error
Algebraic	arc	16.932	−33.350	67.464	67.96	0.069
Geometric	1st arc	16.942	−33.421	67.444	66.98	0.068
	2nd arc	16.922	−33.371	67.454		

**Table 4:** Bean curd thickness measurement results (thickness was measured at θ = −40${}^{\circ }$, −30${}^{\circ }$, −20${}^{\circ }$, −10${}^{\circ }$, 0${}^{\circ }$, 10${}^{\circ }$, 20${}^{\circ }$ and 30${}^{\circ }$ scanning points and averaged).

Measuring Condition	Average of θ = −40${}^{\circ }$∼30${}^{\circ }$			Std. Deviation of θ = −40${}^{\circ }$∼30${}^{\circ }$		
	1 mm	3 mm	5 mm	1 mm	3 mm	5 mm
Operator #1	12.64	12.80	12.84	0.31	0.28	0.24
Operator #2	12.54	12.74	12.74	0.33	0.30	0.30
Operator #3	12.59	12.74	12.73	0.38	0.39	0.34
Operator #4	12.56	12.73	12.74	0.39	0.38	0.36
Operator #5	12.57	12.73	12.77	0.35	0.36	0.31
Average	12.58	12.75	12.77	0.35	0.34	0.31

**Figure A1:**
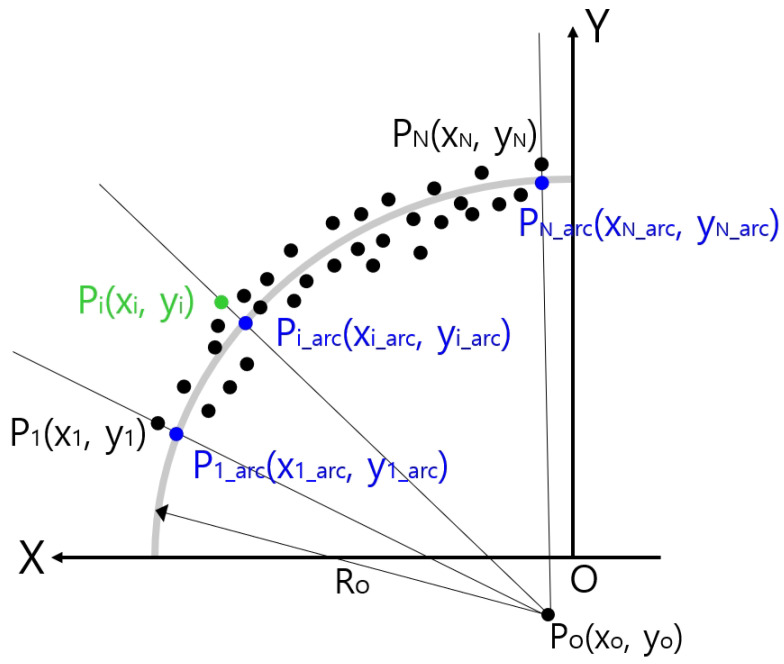
Variables for geometric piecewise arc curve fitting.

## Appendix A. Geometric Piecewise Arc Curve Fitting Details

The inputs to the geometric piecewise arc curve fitting are P${}_{o}$(x${}_{o}$, y${}_{o}$) and R${}_{o}$, as depicted in Figure 3, and the variables for geometric piecewise arc curve fitting are represented in Figure A1, in which P${}_{o}$(x${}_{o}$, y${}_{o}$) and R${}_{o}$ are the center point and radius of the algebraic piecewise arc curve fitting. The gray arc in Figure A1 is the arc drawn by P${}_{o}$(x${}_{o}$, y${}_{o}$) and R${}_{o}$, and the points in Figure A1 constitute a 2D point cloud, which is enumerated by P${}_{1}$(x${}_{1}$, y${}_{1}$), P${}_{2}$(x${}_{2}$, y${}_{2}$)… P${}_{N}$(x${}_{N}$, y${}_{N}$). N is the total number of points in each arc. The blue points in Figure A1 are the projected points on the arc from each point of the point cloud and are denoted as P${}_{i\_arc}$(x${}_{i\_arc}$, y${}_{i\_arc}$). Note that the distance between P${}_{i}$(x${}_{i}$, y${}_{i}$) and the arc is the same as the distance between P${}_{i}$(x${}_{i}$, y${}_{i}$) and P${}_{i\_arc}$(x${}_{i\_arc}$, y${}_{i\_arc}$).

(A1) $$\begin{array}{c} A_{i}=\left(x_{o}-\frac{y_{i}-y_{o}}{x_{i}-x_{o}}\left(\left(y_{i}-y_{o}\right)-\frac{y_{i}-y_{o}}{x_{i}-x_{o}}x_{i}\right)\right)\\ \pm \sqrt{\begin{array}{c} {\left(-x_{o}+\frac{y_{i}-y_{o}}{x_{i}-x_{o}}\left(\left(y_{i}-y_{o}\right)-\frac{y_{i}-y_{o}}{x_{i}-x_{o}}x_{i}\right)\right)}^{2}\\ -\left(1+{\left(\frac{y_{i}-y_{o}}{x_{i}-x_{o}}\right)}^{2}\right)\left(x_{o}^{2}+{\left(\left(y_{i}-y_{o}\right)-\frac{y_{i}-y_{o}}{x_{i}-x_{o}}x_{i}\right)}^{2}-R_{o}^{2}\right) \end{array}} \end{array}$$

(A2) $$B_{i}=1+{\left(\frac{y_{i}-y_{o}}{x_{i}-x_{o}}\right)}^{2}$$

(A3) $$\begin{array}{c} C_{i}={\left(-x_{o}+\frac{y_{i}-y_{o}}{x_{i}-x_{o}}\left(\left(y_{i}-y_{o}\right)-\frac{y_{i}-y_{o}}{x_{i}-x_{o}}x_{i}\right)\right)}^{2}\\ -\left(1+{\left(\frac{y_{i}-y_{o}}{x_{i}-x_{o}}\right)}^{2}\right)\left(x_{o}^{2}+{\left(\left(y_{i}-y_{o}\right)-\frac{y_{i}-y_{o}}{x_{i}-x_{o}}x_{i}\right)}^{2}-R_{o}^{2}\right) \end{array}$$

(A4) $$\left[\begin{array}{c} x_{i\_arc}\\ y_{i\_arc} \end{array}\right]=\left[\begin{array}{c} \frac{A_{i}}{B_{i}}\\ \frac{y_{i}-y_{o}}{x_{i}-x_{o}}\frac{A_{i}}{B_{i}}+y_{i}-\frac{y_{i}-y_{o}}{x_{i}-x_{o}}x_{i} \end{array}\right]$$

Equation (A4) represents the P${}_{i\_arc}$(x${}_{i\_arc}$, y${}_{i\_arc}$) point vector, which is acquired by calculating cross points between line P${}_{o}$P${}_{i}$ and the arc. Note that $A_{i}$, $B_{i}$ and $C_{i}$ are just variables for compactness of equations, and ± in (A1) represents whether points are located in the first quadrant or second quadrant in Figure A1.

(A5) $$e=\sum_{i=1}^{N}({\left(x_{i}-x_{i\_arc}\right)}^{2}+{\left(y_{i}-y_{i\_arc}\right)}^{2})$$

Equation (A5) represents the net arc fitting error. The geometric fitting method by steepest descent algorithm makes use of the partial derivative of e in Equation (A5) with respect to x${}_{o}$, y${}_{o}$ and R${}_{o}$; and increments x${}_{o}$, y${}_{o}$ and R${}_{o}$ in adverse gradient directions.

Equation (A6) represents a partial derivative of e with respect to x${}_{o}$. The partial derivatives of x${}_{i\_arc}$ and y${}_{i\_arc}$ with respect to x${}_{o}$ in Equation (A6) can be calculated by Equations (A7)–(A10), in which the chain rule is used.

(A6) $$\begin{array}{c} \frac{\partial e}{\partial x_{o}}=\frac{\partial }{\partial x_{o}}\left(\sum_{i=1}^{N}({\left(x_{i}-x_{i\_arc}\right)}^{2}+{\left(y_{i}-y_{i\_arc}\right)}^{2})\right)\\ =\sum_{i=1}^{N}\frac{\partial }{\partial x_{o}}\left({\left(x_{i}-x_{i\_arc}\right)}^{2}+{\left(y_{i}-y_{i\_arc}\right)}^{2}\right)\\ =-2\sum_{i=1}^{N}\left(\left(x_{i}-x_{i\_arc}\right)\frac{\partial x_{i\_arc}}{\partial x_{o}}+\left(y_{i}-y_{i\_arc}\right)\frac{\partial y_{i\_arc}}{\partial x_{o}}\right) \end{array}$$

(A7) $$\frac{\partial x_{i\_arc}}{\partial x_{o}}=\frac{\partial }{\partial x_{o}}\left(\frac{A_{i}}{B_{i}}\right)=\frac{\frac{\partial A_{i}}{\partial x_{o}}B_{i}-A_{i}\frac{\partial B_{i}}{\partial x_{o}}}{B_{i}^{2}}$$

(A8) $$\begin{array}{c} \frac{\partial A_{i}}{\partial x_{o}}=1-\frac{y_{i}-y_{o}}{{\left(x_{i}-x_{o}\right)}^{2}}\left(\left(y_{i}-y_{o}\right)-\frac{y_{i}-y_{o}}{x_{i}-x_{o}}x_{i}\right)+\frac{{\left(y_{i}-y_{o}\right)}^{2}}{{\left(x_{i}-x_{o}\right)}^{3}}x_{i}\\ \pm \frac{1}{\sqrt{C_{i}}}\left(-x_{o}+\frac{y_{i}-y_{o}}{x_{i}-x_{o}}\left(\left(y_{i}-y_{o}\right)-\frac{y_{i}-y_{o}}{x_{i}-x_{o}}x_{i}\right)\right)\\ \left(-1+\frac{y_{i}-y_{o}}{{\left(x_{i}-x_{o}\right)}^{2}}\left(\left(y_{i}-y_{o}\right)-\frac{y_{i}-y_{o}}{x_{i}-x_{o}}x_{i}\right)-\frac{{\left(y_{i}-y_{o}\right)}^{2}}{{\left(x_{i}-x_{o}\right)}^{3}}x_{i}\right)\\ \mp \frac{1}{\sqrt{C_{i}}}\frac{{\left(y_{i}-y_{o}\right)}^{2}}{{\left(x_{i}-x_{o}\right)}^{3}}\left(x_{o}^{2}+{\left(\left(y_{i}-y_{o}\right)-\frac{y_{i}-y_{o}}{x_{i}-x_{o}}x_{i}\right)}^{2}-R_{o}^{2}\right)\\ \mp \frac{1}{\sqrt{C_{i}}}\left(1+{\left(\frac{y_{i}-y_{o}}{x_{i}-x_{o}}\right)}^{2}\right)\left(x_{o}-x_{i}\frac{y_{i}-y_{o}}{{\left(x_{i}-x_{o}\right)}^{2}}\left(\left(y_{i}-y_{o}\right)-\frac{y_{i}-y_{o}}{x_{i}-x_{o}}x_{i}\right)\right) \end{array}$$

(A9) $$\frac{\partial B_{i}}{\partial x_{o}}=2\frac{{\left(y_{i}-y_{o}\right)}^{2}}{{\left(x_{i}-x_{o}\right)}^{3}}$$

(A10) $$\frac{\partial y_{i\_arc}}{\partial x_{o}}=\frac{y_{i}-y_{o}}{{\left(x_{i}-x_{o}\right)}^{2}}\frac{A_{i}}{B_{i}}+\frac{y_{i}-y_{o}}{x_{i}-x_{o}}\frac{\frac{\partial A_{i}}{\partial x_{o}}B_{i}-A_{i}\frac{\partial B_{i}}{\partial x_{o}}}{B_{i}^{2}}-x_{i}\frac{y_{i}-y_{o}}{{\left(x_{i}-x_{o}\right)}^{2}}$$

Equation (A11) represents the partial derivative of e with respect to y${}_{o}$. The partial derivatives of x${}_{i\_arc}$ and y${}_{i\_arc}$ with respect to y${}_{o}$ in Equation (A11) can be calculated by Equations (A12)–(A15).

(A11) $$\frac{\partial e}{\partial y_{o}}=-2\sum_{i=1}^{N}\left(\left(x_{i}-x_{i\_arc}\right)\frac{\partial x_{i\_arc}}{\partial y_{o}}+\left(y_{i}-y_{i\_arc}\right)\frac{\partial y_{i\_arc}}{\partial y_{o}}\right)$$

(A12) $$\frac{\partial x_{i\_arc}}{\partial y_{o}}=\frac{\partial }{\partial y_{o}}\left(\frac{A_{i}}{B_{i}}\right)=\frac{\frac{\partial A_{i}}{\partial y_{o}}B_{i}-A_{i}\frac{\partial B_{i}}{\partial y_{o}}}{B_{i}^{2}}$$

(A13) $$\begin{array}{c} \frac{\partial A_{i}}{\partial y_{o}}=\frac{1}{x_{i}-x_{o}}\left(\left(y_{i}-y_{o}\right)-\frac{y_{i}-y_{o}}{x_{i}-x_{o}}x_{i}\right)\\ \pm \frac{1}{\sqrt{C_{i}}}\left(-x_{o}+\frac{y_{i}-y_{o}}{x_{i}-x_{o}}\left(\left(y_{i}-y_{o}\right)-\frac{y_{i}-y_{o}}{x_{i}-x_{o}}x_{i}\right)\right)\\ \left(-\frac{1}{x_{i}-x_{o}}\left(\left(y_{i}-y_{o}\right)-\frac{y_{i}-y_{o}}{x_{i}-x_{o}}x_{i}\right)+\frac{y_{i}-y_{o}}{x_{i}-x_{o}}\left(-1+\frac{x_{i}}{x_{i}-x_{o}}\right)\right)\\ \pm \frac{1}{\sqrt{C_{i}}}\frac{y_{i}-y_{o}}{{\left(x_{i}-x_{o}\right)}^{2}}\left(x_{o}^{2}+{\left(\left(y_{i}-y_{o}\right)-\frac{y_{i}-y_{o}}{x_{i}-x_{o}}x_{i}\right)}^{2}-R_{o}^{2}\right)\\ \mp \frac{1}{2\sqrt{C_{i}}}\left(1+{\left(\frac{y_{i}-y_{o}}{x_{i}-x_{o}}\right)}^{2}\right)\left(2\left(\left(y_{i}-y_{o}\right)-\frac{y_{i}-y_{o}}{x_{i}-x_{o}}x_{i}\right)\left(-1+\frac{x_{i}}{x_{i}-x_{o}}\right)\right) \end{array}$$

(A14) $$\frac{\partial B_{i}}{\partial y_{o}}=-2\frac{y_{i}-y_{o}}{{\left(x_{i}-x_{o}\right)}^{2}}$$

(A15) $$\frac{\partial y_{i\_arc}}{\partial y_{o}}=-\frac{1}{x_{i}-x_{o}}\frac{A_{i}}{B_{i}}+\frac{y_{i}-y_{o}}{x_{i}-x_{o}}\frac{\frac{\partial A_{i}}{\partial y_{o}}B_{i}-A_{i}\frac{\partial B_{i}}{\partial y_{o}}}{B_{i}^{2}}+\frac{x_{i}}{x_{i}-x_{o}}$$

Equation (A16) represents the partial derivative of e with respect to y${}_{o}$. The partial derivatives of x${}_{i\_arc}$ and y${}_{i\_arc}$ with respect to y${}_{o}$ in Equation (A16) can be calculated by Equations (A17) and (A18).

(A16) $$\frac{\partial e}{\partial R_{o}}=-2\sum_{i=1}^{N}\left(\left(x_{i}-x_{i\_arc}\right)\frac{\partial x_{i\_arc}}{\partial R_{o}}+\left(y_{i}-y_{i\_arc}\right)\frac{\partial y_{i\_arc}}{\partial R_{o}}\right)$$

(A17) $$\frac{\partial x_{i\_arc}}{\partial R_{o}}=\pm \frac{R_{o}}{\sqrt{C_{i}}}$$

(A18) $$\frac{\partial y_{i\_arc}}{\partial R_{o}}=\pm \frac{y_{i}-y_{o}}{x_{i}-x_{o}}\frac{\partial x_{i\_arc}}{\partial R_{o}}=\pm \frac{y_{i}-y_{o}}{x_{i}-x_{o}}\frac{R_{o}}{\sqrt{C_{i}}}$$

The incremental values Δx${}_{o}$, Δy${}_{o}$ and ΔR${}_{o}$ are calculated by Equation (A19). The diagonal element in the matrix in Equation (A19) represents the adverse normalized gradient directions of e with respect to x${}_{o}$, y${}_{o}$ and R${}_{o}$. The 0.01, 0.01 and 0.1 mm values in Equation (A19) are determined by heuristic method.

(A19) $$\left[\begin{array}{c} \Delta x_{o}\\ \Delta y_{o}\\ \Delta R_{o} \end{array}\right]=\left[\begin{array}{ccc} \frac{-\frac{\partial e}{\partial x_{o}}}{{∥\frac{\partial e}{\partial x_{o}}∥}_{2}} & 0 & 0\\ 0 & \frac{-\frac{\partial e}{\partial y_{o}}}{{∥\frac{\partial e}{\partial y_{o}}∥}_{2}} & 0\\ 0 & 0 & \frac{-\frac{\partial e}{\partial R_{o}}}{{∥\frac{\partial e}{\partial R_{o}}∥}_{2}} \end{array}\right]\left[\begin{array}{c} 0.01\\ 0.01\\ 0.1 \end{array}\right]$$

If Δx${}_{o}$, Δy${}_{o}$ and ΔR${}_{o}$ are determined, x${}_{o}$, y${}_{o}$ and R${}_{o}$ are updated by Equation (A20), and the calculation iterates from Equations (A1)–(A20) until the epoch reaches 500 or the gradient value is less than the threshold.

(A20) $$\left[\begin{array}{c} x_{o}\\ y_{o}\\ R_{o} \end{array}\right]=\left[\begin{array}{c} x_{o}+\Delta x_{o}\\ y_{o}+\Delta y_{o}\\ R_{o}+\Delta R_{o} \end{array}\right]$$
